# 2-Amino-4-(4-hy­droxy-3,5-dimeth­oxy­phen­yl)-6-phenyl­nicotinonitrile

**DOI:** 10.1107/S1600536810039127

**Published:** 2010-10-09

**Authors:** Xiao-Hui Yang, Yong-Hong Zhou, Li-Hong Hu, Hong-Jun Liu

**Affiliations:** a Institute of Chemical Industry of Forest Products, Chinese Academy of Forestry, Nanjing 210042, People’s Republic of China

## Abstract

In the title compound, C_20_H_17_N_3_O_3_, the dihedral angles between the central pyridine ring and the two terminal rings are 15.07 (3) and 43.24 (3)°. The dihedral angle between the two terminal rings is 37.49 (4)° In the crystal, inter­molecular amine N—H⋯N_nitrile_ hydrogen-bonding inter­actions form inversion dimers, which are linked into chains through amine N—H⋯O_meth­oxy_ hydrogen bonds.

## Related literature

For literature on the biological applications of nicotine derivatives, see Hökelek & Necefouglu (1996 ▶, 1999 ▶). For literature on mol­ecules containing the cyano­pyridine moiety and their ability to act as ligands towards transition metal ions and new drugs, see: Alyoubi (2000 ▶); Desai & Shah (2003 ▶); Murata *et al.* (2004 ▶). For a related structure, see: Fun *et al.* (1996 ▶).
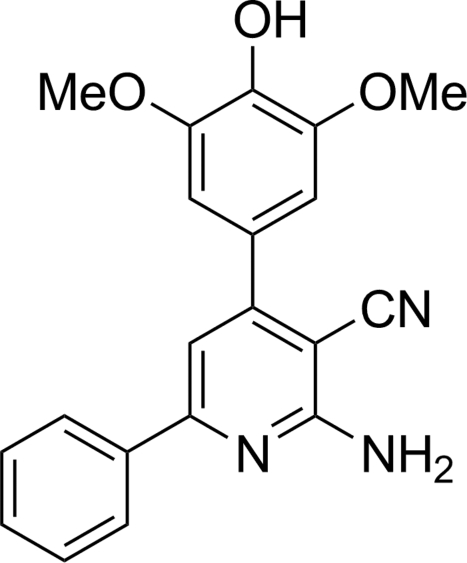


## Experimental

### 

#### Crystal data

C_20_H_17_N_3_O_3_
*M* *_r_* = 347.37
Triclinic, 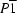

*a* = 8.1320 (16) Å
*b* = 10.497 (2) Å
*c* = 10.914 (2) Å
α = 77.28 (3)°
β = 68.36 (3)°
γ = 84.66 (3)°
*V* = 844.6 (3) Å^3^
*Z* = 2
Mo *K*α radiation
μ = 0.09 mm^−1^
*T* = 293 K
0.20 × 0.10 × 0.10 mm

#### Data collection

Enraf–Nonius CAD-4 four-circle diffractometer
Absorption correction: ψ scan (semi-empirical, using intensity measurements; North *et al.*, 1968 ▶) *T*_min_ = 0.981, *T*_max_ = 0.991
3294 measured reflections
3058 independent reflections
1776 reflections with *I* > 2σ(*I*)
*R*_int_ = 0.031
3 standard reflections every 200 reflections  intensity decay: 1%

#### Refinement

*R*[*F* ^2^ > 2σ(*F* ^2^)] = 0.069
*wR*(*F* ^2^) = 0.169
*S* = 1.01
3058 reflections
235 parameters
H-atom parameters constrained
Δρ_max_ = 0.37 e Å^−3^
Δρ_min_ = −0.25 e Å^−3^

### 

Data collection: *SMART* (Bruker, 2004 ▶); cell refinement: *SMART*; data reduction: *SAINT-Plus* (Bruker, 2004 ▶); program(s) used to solve structure: *SHELXS97* (Sheldrick, 2008 ▶); program(s) used to refine structure: *SHELXL97* (Sheldrick, 2008 ▶); molecular graphics: *SHELXTL* (Sheldrick, 2008 ▶); software used to prepare material for publication: *SHELXTL*.

## Supplementary Material

Crystal structure: contains datablocks I, global. DOI: 10.1107/S1600536810039127/zs2068sup1.cif

Structure factors: contains datablocks I. DOI: 10.1107/S1600536810039127/zs2068Isup2.hkl

Additional supplementary materials:  crystallographic information; 3D view; checkCIF report

## Figures and Tables

**Table 1:** Hydrogen-bond geometry (Å, °)

*D*—H⋯*A*	*D*—H	H⋯*A*	*D*⋯*A*	*D*—H⋯*A*
N2—H2*A*⋯O2^i^	0.86	2.27	3.101 (4)	162
N2—H2*B*⋯N3^ii^	0.86	2.31	3.098 (5)	152

Symmetry codes: (i) 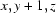; (ii) 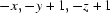.

**Fig. 1.**
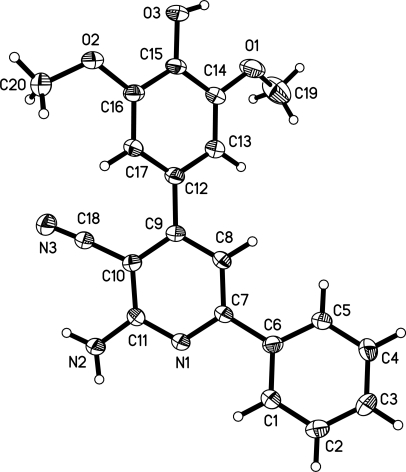
Molecular structure of the title compound, showing the atom-numbering scheme. Displacement ellipsoids are drawn at the 50% probability level.

**Fig. 2.**
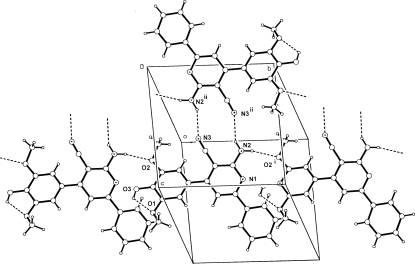
The packing of the title compound, viewed along the a axis of the unit cell. Dashed lines indicate hydrogen bonds. For symmetry codes, see Table 1.

## supplementary crystallographic information

### Comment

Nicotine derivatives have a wide range of biological applications. Niacin is a vitamin that contains nicotinamide, deficiency of which makes the body lose copper, thereby giving rise to the pellagra disease (Hökelek & Necefouglu, 1999). The nicotinic acid derivative *N*,*N*-diethylnicotinamide, which is commonly known as DENA, has a respiratory stimulating property (Hökelek & Necefouglu, 1996). In addition, it has been demonstrated that molecules containing the cyanopyridine moiety may be able to act as ligands towards transition-metal ions (Alyoubi, 2000), new drugs (Murata *et al.*, 2004; Desai & Shah, 2003) and significant intermediates for the synthesis of important vitamins such as nicotinic acids and nicotinamides. For these reasons, the synthesis of new derived cyanopyridine compounds is strongly desired. Against this background and in order to obtain detailed information on molecular conformation in the solid state, the X-ray study of the title compound C_20_H_17_N_3_O_3_ (I) was carried out and the results are presented here.

In the molecular structure of (I) (Fig. 1), the pyridine ring is almost planar, with a maximum deviation from the plane of 0.031 (5) Å for C10, and it forms a dihedral angle of 15.07 (3)° with the mean plane through benzene ring and another dihedral angle of 43.24 (3)° with the mean plane through the 4-hydroxy-3,5-dimethoxy-substituted benzene ring. The hydroxy group gives an interaction with a methoxy-O acceptor [2.654 (4) Å]. The dihedral angle between the planes of the pyridine and the second phenyl rings [15.07 (3)°] is slightly larger than that reported for a related structure [9.04 (6)°] (Fun *et al.*, 1996). In (I) the ring conformation is stabilized by the presence of a short intramolecular aromatic ring C1—H···N1_pyridine_ interaction [2.790 (5) Å]. The methoxy substituent groups lie slightly out of plane of the benzene ring [torsion angles C20—O2—C16—C17, -18.0 (5)° and C19—O1—C14—C13, 27.2 (7)°]. The crystal packing of the title compound is stabilized by intermolecular amine N—H···N_nitrile_ hydrogen-bonding interactions forming centrosymmetric cyclic dimers which are linked through amine N—H···O_methoxy_ hydrogen bonds into one-dimensional chains which extend along the *b* cell direction (Fig. 2).

### Experimental

To a refluxing solution of acetophenone (2 mmol) in ethanol (10 ml), malononitrile (2 mmol), 4-hydroxy-3,5-dimethoxybenzaldehyde (syringaldehyde) (2 mmol) and ammonium acetate (2 mmol) were added, and the resulting solution was refluxed for 6 h. The solvent was distilled off under reduced pressure and the resulting residue was purified by column chromatography using silica gel eluent (100–200 mesh). Single crystals were obtained by slow evaporation using a petroleum ether/ethyl acetate (1: 3) solvent system.

### Refinement

The H atoms were fixed geometrically and allowed to ride on the attached non-H atoms, with O—H = 0.82 Å, N—H = 0.86 Å and C—H = 0.93–0.96 Å, and with *U*_iso_(H)= 1.5 *U*_eq_(C) for methyl H atoms and 1.2 *U*_eq_(C) for all other atoms.

### Crystal data

**Table:**

C_20_H_17_N_3_O_3_	*Z* = 2
*M**_r_* = 347.37	*F*(000) = 364
Triclinic, *P*1	*D*_x_ = 1.366 Mg m^−^^3^
Hall symbol: -P 1	Mo *K*α radiation, λ = 0.71073 Å
*a* = 8.1320 (16) Å	Cell parameters from 25 reflections
*b* = 10.497 (2) Å	θ = 9–12°
*c* = 10.914 (2) Å	µ = 0.09 mm^−^^1^
α = 77.28 (3)°	*T* = 293 K
β = 68.36 (3)°	Block, colourless
γ = 84.66 (3)°	0.20 × 0.10 × 0.10 mm
*V* = 844.6 (3) Å^3^	

### Data collection

**Table:**

Enraf–Nonius CAD-4 four-circle diffractometer	1776 reflections with *I* > 2σ(*I*)
Radiation source: fine-focus sealed tube	*R*_int_ = 0.031
graphite	θ_max_ = 25.3°, θ_min_ = 2.0°
ω/2θ scans	*h* = 0→9
Absorption correction: ψ scan (semi-empirical (using intensity measurements); North *et al.*, 1968)	*k* = −12→12
*T*_min_ = 0.981, *T*_max_ = 0.991	*l* = −12→13
3294 measured reflections	3 standard reflections every 200 reflections
3058 independent reflections	intensity decay: 1%

### Refinement

**Table:**

Refinement on *F*^2^	Primary atom site location: structure-invariant direct methods
Least-squares matrix: full	Secondary atom site location: difference Fourier map
*R*[*F*^2^ > 2σ(*F*^2^)] = 0.069	Hydrogen site location: inferred from neighbouring sites
*wR*(*F*^2^) = 0.169	H-atom parameters constrained
*S* = 1.01	*w* = 1/[σ^2^(*F*_o_^2^) + (0.06*P*)^2^ + 0.5*P*] where *P* = (*F*_o_^2^ + 2*F*_c_^2^)/3
3058 reflections	(Δ/σ)_max_ < 0.001
235 parameters	Δρ_max_ = 0.37 e Å^−^^3^
0 restraints	Δρ_min_ = −0.25 e Å^−^^3^

### Special details

**Table:**

Geometry. Bond distances, angles etc. have been calculated using the rounded fractional coordinates. All su's are estimated from the variances of the (full) variance-covariance matrix. The cell esds are taken into account in the estimation of distances, angles and torsion angles
Refinement. Refinement of *F*^2^ against ALL reflections. The weighted *R*-factor *wR* and goodness of fit *S* are based on *F*^2^, conventional *R*-factors *R* are based on *F*, with *F* set to zero for negative *F*^2^. The threshold expression of *F*^2^ > σ(*F*^2^) is used only for calculating *R*-factors(gt) *etc*. and is not relevant to the choice of reflections for refinement. *R*-factors based on *F*^2^ are statistically about twice as large as those based on *F*, and *R*- factors based on ALL data will be even larger.

### Fractional atomic coordinates and isotropic or equivalent isotropic displacement parameters (Å^2^)

**Table:**

	*x*	*y*	*z*	*U*_iso_*/*U*_eq_	
O1	0.2727 (5)	−0.1198 (2)	1.0345 (3)	0.0793 (13)	
O2	0.2820 (4)	−0.0923 (2)	0.5979 (3)	0.0590 (10)	
O3	0.2628 (3)	−0.2323 (2)	0.8421 (2)	0.0535 (9)	
N1	0.4176 (4)	0.5786 (2)	0.6850 (3)	0.0442 (10)	
N2	0.2265 (4)	0.6279 (3)	0.5721 (3)	0.0598 (11)	
N3	0.0259 (5)	0.3444 (3)	0.5868 (4)	0.0697 (14)	
C1	0.6885 (5)	0.6715 (3)	0.7396 (4)	0.0529 (12)	
C2	0.8049 (5)	0.7227 (4)	0.7807 (4)	0.0641 (16)	
C3	0.8678 (5)	0.6491 (4)	0.8747 (4)	0.0630 (16)	
C4	0.8153 (5)	0.5217 (4)	0.9273 (4)	0.0600 (14)	
C5	0.6991 (5)	0.4705 (3)	0.8868 (4)	0.0512 (11)	
C6	0.6330 (4)	0.5439 (3)	0.7930 (3)	0.0433 (11)	
C7	0.5053 (4)	0.4907 (3)	0.7504 (3)	0.0413 (11)	
C8	0.4765 (5)	0.3580 (3)	0.7747 (3)	0.0459 (11)	
C9	0.3556 (4)	0.3108 (3)	0.7332 (3)	0.0429 (11)	
C10	0.2667 (4)	0.4028 (3)	0.6648 (3)	0.0436 (11)	
C11	0.3040 (4)	0.5356 (3)	0.6404 (3)	0.0423 (11)	
C12	0.3250 (4)	0.1688 (3)	0.7618 (4)	0.0453 (11)	
C13	0.3115 (5)	0.0937 (3)	0.8868 (4)	0.0528 (14)	
C14	0.2894 (5)	−0.0402 (3)	0.9136 (4)	0.0496 (11)	
C15	0.2843 (4)	−0.1017 (3)	0.8137 (3)	0.0395 (11)	
C16	0.2939 (4)	−0.0246 (3)	0.6902 (3)	0.0428 (11)	
C17	0.3166 (4)	0.1081 (3)	0.6633 (3)	0.0438 (11)	
C18	0.1322 (5)	0.3671 (3)	0.6226 (4)	0.0477 (11)	
C19	0.2036 (7)	−0.0690 (5)	1.1506 (5)	0.096 (2)	
C20	0.2425 (5)	−0.0191 (4)	0.4867 (4)	0.0641 (16)	
H1B	0.64720	0.72290	0.67560	0.0630*	
H2A	0.25100	0.70880	0.55940	0.0720*	
H2B	0.15250	0.60600	0.54110	0.0720*	
H2C	0.84120	0.80870	0.74410	0.0770*	
H3A	0.94520	0.68490	0.90260	0.0750*	
H3B	0.25970	−0.26200	0.91900	0.0800*	
H4A	0.85840	0.47030	0.99020	0.0720*	
H5A	0.66400	0.38430	0.92330	0.0620*	
H8A	0.53890	0.29950	0.81940	0.0550*	
H13A	0.31720	0.13370	0.95320	0.0640*	
H17A	0.32640	0.15730	0.57890	0.0520*	
H19A	0.20010	−0.13640	1.22690	0.1430*	
H19B	0.08590	−0.03610	1.16140	0.1430*	
H19C	0.27690	0.00070	1.14390	0.1430*	
H20A	0.23850	−0.07660	0.43050	0.0970*	
H20B	0.33230	0.04490	0.43580	0.0970*	
H20C	0.12980	0.02400	0.51810	0.0970*	

### Atomic displacement parameters (Å^2^)

**Table:**

	*U*^11^	*U*^22^	*U*^33^	*U*^12^	*U*^13^	*U*^23^
O1	0.144 (3)	0.0382 (15)	0.0645 (19)	0.0084 (16)	−0.051 (2)	−0.0079 (13)
O2	0.092 (2)	0.0321 (13)	0.0648 (17)	−0.0016 (13)	−0.0419 (16)	−0.0090 (12)
O3	0.0714 (17)	0.0284 (12)	0.0605 (16)	−0.0096 (11)	−0.0277 (14)	0.0025 (11)
N1	0.0520 (18)	0.0311 (14)	0.0546 (18)	−0.0009 (12)	−0.0285 (15)	−0.0023 (13)
N2	0.078 (2)	0.0320 (16)	0.088 (2)	−0.0033 (15)	−0.057 (2)	−0.0006 (15)
N3	0.079 (2)	0.055 (2)	0.098 (3)	−0.0013 (17)	−0.060 (2)	−0.0120 (19)
C1	0.059 (2)	0.043 (2)	0.061 (2)	−0.0062 (17)	−0.033 (2)	0.0034 (17)
C2	0.069 (3)	0.042 (2)	0.086 (3)	−0.0160 (19)	−0.038 (2)	0.002 (2)
C3	0.054 (2)	0.062 (3)	0.083 (3)	−0.015 (2)	−0.036 (2)	−0.009 (2)
C4	0.055 (2)	0.057 (2)	0.075 (3)	−0.0006 (19)	−0.040 (2)	0.002 (2)
C5	0.056 (2)	0.0345 (18)	0.067 (2)	−0.0051 (16)	−0.032 (2)	0.0010 (17)
C6	0.040 (2)	0.0341 (17)	0.056 (2)	−0.0037 (15)	−0.0184 (17)	−0.0061 (16)
C7	0.047 (2)	0.0307 (17)	0.048 (2)	0.0006 (15)	−0.0227 (17)	−0.0021 (15)
C8	0.053 (2)	0.0314 (17)	0.061 (2)	0.0061 (15)	−0.0336 (19)	−0.0043 (16)
C9	0.053 (2)	0.0290 (17)	0.051 (2)	0.0014 (15)	−0.0240 (18)	−0.0082 (15)
C10	0.049 (2)	0.0335 (17)	0.053 (2)	−0.0024 (15)	−0.0237 (18)	−0.0080 (15)
C11	0.048 (2)	0.0345 (18)	0.049 (2)	0.0027 (15)	−0.0250 (18)	−0.0056 (15)
C12	0.049 (2)	0.0311 (17)	0.061 (2)	0.0032 (15)	−0.0280 (19)	−0.0067 (16)
C13	0.069 (3)	0.0368 (19)	0.063 (2)	0.0020 (17)	−0.037 (2)	−0.0086 (17)
C14	0.064 (2)	0.0323 (18)	0.056 (2)	0.0017 (16)	−0.030 (2)	−0.0019 (17)
C15	0.0369 (19)	0.0266 (16)	0.051 (2)	−0.0017 (13)	−0.0140 (16)	−0.0021 (15)
C16	0.043 (2)	0.0313 (17)	0.055 (2)	0.0010 (14)	−0.0181 (17)	−0.0102 (16)
C17	0.050 (2)	0.0322 (17)	0.051 (2)	−0.0021 (15)	−0.0241 (18)	−0.0011 (15)
C18	0.060 (2)	0.0331 (18)	0.058 (2)	0.0024 (16)	−0.032 (2)	−0.0077 (16)
C19	0.113 (4)	0.086 (4)	0.071 (3)	0.020 (3)	−0.026 (3)	−0.001 (3)
C20	0.077 (3)	0.057 (2)	0.075 (3)	0.000 (2)	−0.048 (2)	−0.011 (2)

### Geometric parameters (Å, °)

**Table:**

O1—C14	1.364 (5)	C10—C11	1.401 (5)
O1—C19	1.387 (6)	C10—C18	1.440 (5)
O2—C16	1.389 (4)	C12—C17	1.389 (5)
O2—C20	1.413 (5)	C12—C13	1.388 (5)
O3—C15	1.350 (4)	C13—C14	1.386 (5)
O3—H3B	0.8200	C14—C15	1.398 (5)
N1—C7	1.354 (4)	C15—C16	1.390 (4)
N1—C11	1.342 (5)	C16—C17	1.374 (5)
N2—C11	1.344 (5)	C1—H1B	0.9300
N3—C18	1.134 (6)	C2—H2C	0.9300
N2—H2B	0.8600	C3—H3A	0.9300
N2—H2A	0.8600	C4—H4A	0.9300
C1—C2	1.378 (6)	C5—H5A	0.9300
C1—C6	1.383 (5)	C8—H8A	0.9300
C2—C3	1.370 (6)	C13—H13A	0.9300
C3—C4	1.374 (6)	C17—H17A	0.9300
C4—C5	1.373 (6)	C19—H19A	0.9600
C5—C6	1.383 (5)	C19—H19B	0.9600
C6—C7	1.478 (5)	C19—H19C	0.9600
C7—C8	1.385 (5)	C20—H20A	0.9600
C8—C9	1.391 (5)	C20—H20B	0.9600
C9—C10	1.401 (5)	C20—H20C	0.9600
C9—C12	1.479 (5)		
			
C14—O1—C19	119.3 (3)	O3—C15—C16	122.4 (3)
C16—O2—C20	117.4 (3)	C14—C15—C16	118.3 (3)
C15—O3—H3B	109.00	O2—C16—C15	114.9 (3)
C7—N1—C11	119.1 (3)	C15—C16—C17	121.4 (3)
C11—N2—H2B	120.00	O2—C16—C17	123.7 (3)
H2A—N2—H2B	120.00	C12—C17—C16	120.1 (3)
C11—N2—H2A	120.00	N3—C18—C10	177.1 (4)
C2—C1—C6	120.3 (4)	C2—C1—H1B	120.00
C1—C2—C3	121.1 (4)	C6—C1—H1B	120.00
C2—C3—C4	119.2 (4)	C1—C2—H2C	119.00
C3—C4—C5	119.9 (4)	C3—C2—H2C	119.00
C4—C5—C6	121.7 (3)	C2—C3—H3A	120.00
C5—C6—C7	122.2 (3)	C4—C3—H3A	120.00
C1—C6—C5	117.9 (3)	C3—C4—H4A	120.00
C1—C6—C7	119.9 (3)	C5—C4—H4A	120.00
C6—C7—C8	122.3 (3)	C4—C5—H5A	119.00
N1—C7—C8	121.2 (3)	C6—C5—H5A	119.00
N1—C7—C6	116.5 (3)	C7—C8—H8A	120.00
C7—C8—C9	121.0 (3)	C9—C8—H8A	119.00
C8—C9—C10	117.2 (3)	C12—C13—H13A	120.00
C8—C9—C12	120.1 (3)	C14—C13—H13A	120.00
C10—C9—C12	122.7 (3)	C12—C17—H17A	120.00
C9—C10—C11	119.3 (3)	C16—C17—H17A	120.00
C9—C10—C18	122.6 (3)	O1—C19—H19A	109.00
C11—C10—C18	118.0 (3)	O1—C19—H19B	109.00
N1—C11—C10	122.1 (3)	O1—C19—H19C	109.00
N2—C11—C10	122.0 (3)	H19A—C19—H19B	109.00
N1—C11—N2	115.9 (3)	H19A—C19—H19C	109.00
C13—C12—C17	119.2 (3)	H19B—C19—H19C	109.00
C9—C12—C13	119.9 (3)	O2—C20—H20A	109.00
C9—C12—C17	120.9 (3)	O2—C20—H20B	110.00
C12—C13—C14	120.6 (3)	O2—C20—H20C	110.00
O1—C14—C15	116.0 (3)	H20A—C20—H20B	109.00
C13—C14—C15	120.2 (3)	H20A—C20—H20C	109.00
O1—C14—C13	123.8 (3)	H20B—C20—H20C	109.00
O3—C15—C14	119.2 (3)		
			
C19—O1—C14—C13	27.2 (7)	C12—C9—C10—C11	−179.4 (3)
C19—O1—C14—C15	−153.2 (4)	C12—C9—C10—C18	3.1 (5)
C20—O2—C16—C15	163.1 (3)	C8—C9—C12—C13	42.3 (5)
C20—O2—C16—C17	−18.0 (5)	C8—C9—C12—C17	−135.1 (4)
C11—N1—C7—C6	178.0 (3)	C10—C9—C12—C13	−137.6 (4)
C11—N1—C7—C8	−1.6 (5)	C10—C9—C12—C17	45.0 (5)
C7—N1—C11—N2	−177.7 (3)	C9—C10—C11—N1	−2.6 (5)
C7—N1—C11—C10	3.1 (5)	C9—C10—C11—N2	178.1 (3)
C6—C1—C2—C3	−0.1 (6)	C18—C10—C11—N1	175.0 (3)
C2—C1—C6—C5	−0.6 (5)	C18—C10—C11—N2	−4.3 (5)
C2—C1—C6—C7	178.7 (3)	C9—C12—C13—C14	−177.6 (4)
C1—C2—C3—C4	0.8 (6)	C17—C12—C13—C14	−0.2 (6)
C2—C3—C4—C5	−1.0 (6)	C9—C12—C17—C16	177.9 (3)
C3—C4—C5—C6	0.3 (6)	C13—C12—C17—C16	0.5 (5)
C4—C5—C6—C1	0.5 (6)	C12—C13—C14—O1	−179.1 (4)
C4—C5—C6—C7	−178.9 (3)	C12—C13—C14—C15	1.4 (6)
C1—C6—C7—N1	−15.1 (5)	O1—C14—C15—O3	0.4 (5)
C1—C6—C7—C8	164.5 (3)	O1—C14—C15—C16	177.6 (4)
C5—C6—C7—N1	164.2 (3)	C13—C14—C15—O3	179.9 (4)
C5—C6—C7—C8	−16.2 (5)	C13—C14—C15—C16	−2.8 (6)
N1—C7—C8—C9	−0.3 (5)	O3—C15—C16—O2	−0.8 (5)
C6—C7—C8—C9	−179.9 (3)	O3—C15—C16—C17	−179.7 (3)
C7—C8—C9—C10	0.8 (5)	C14—C15—C16—O2	−177.9 (3)
C7—C8—C9—C12	−179.2 (3)	C14—C15—C16—C17	3.2 (5)
C8—C9—C10—C11	0.7 (4)	O2—C16—C17—C12	179.2 (3)
C8—C9—C10—C18	−176.8 (3)	C15—C16—C17—C12	−2.0 (5)

### Hydrogen-bond geometry (Å, °)

**Table:**

*D*—H···*A*	*D*—H	H···*A*	*D*···*A*	*D*—H···*A*
N2—H2A···O2^i^	0.86	2.27	3.101 (4)	162
N2—H2B···N3^ii^	0.86	2.31	3.098 (5)	152
O3—H3B···O1	0.82	2.19	2.654 (4)	116
C1—H1B···N1	0.93	2.47	2.790 (5)	100

Symmetry codes: (i) *x*, *y*+1, *z*; (ii) −*x*, −*y*+1, −*z*+1.
